# Next-Generation Sequencing of Apoptotic DNA Breakpoints Reveals Association with Actively Transcribed Genes and Gene Translocations

**DOI:** 10.1371/journal.pone.0026054

**Published:** 2011-11-08

**Authors:** Melissa J. Fullwood, Joanne Lee, Lifang Lin, Guoliang Li, Mikael Huss, Patrick Ng, Wing-Kin Sung, Shirish Shenolikar

**Affiliations:** 1 A*STAR-Duke-NUS Neuroscience Partnership, Duke-NUS Graduate Medical School Singapore, Singapore, Singapore; 2 Genome Institute of Singapore, Agency for Science, Technology and Research (A*STAR), Singapore, Singapore; 3 Department of Biochemistry, National University of Singapore, Singapore, Singapore; 4 1st Base Pte Ltd, Singapore, Singapore; 5 Signature Research Programs in Cardiovascular and Metabolic Disorders and Neuroscience and Behavioral Disorders, Duke-NUS Graduate Medical School Singapore, Singapore, Singapore; 6 Department of Pharmacology and Cancer Biology, Duke University Medical Center, Durham, North Carolina, United States of America; Virginia Commonwealth University, United States of America

## Abstract

DNA fragmentation is a well-recognized hallmark of apoptosis. However, the precise DNA sequences cleaved during apoptosis triggered by distinct mechanisms remain unclear. We used next-generation sequencing of DNA fragments generated in Actinomycin D-treated human HL-60 leukemic cells to generate a high-throughput, global map of apoptotic DNA breakpoints. These data highlighted that DNA breaks are non-random and show a significant association with active genes and open chromatin regions. We noted that transcription factor binding sites were also enriched within a fraction of the apoptotic breakpoints. Interestingly, extensive apoptotic cleavage was noted within genes that are frequently translocated in human cancers. We speculate that the non-random fragmentation of DNA during apoptosis may contribute to gene translocations and the development of human cancers.

## Introduction

Many anti-cancer drugs induce cell death in target cells via programmed cell death or apoptosis [1]. Apoptosis, which is also important in normal tissue development, is significantly activated during many pathological conditions and involves nuclear condensation and fragmentation of genomic DNA and its subsequent engulfment by neighboring cells [2]. A complex network of biochemical pathways trigger or modulate apoptosis in mammalian cells. For example, the activation of a cascade of caspases, a family of thiol proteases, leads to the cleavage of numerous proteins that in turn results in alterations in cell morphology that are characteristic of apoptosis. In addition, the Bcl-2 extended family members, such as BAX, function as inhibitors of apoptosis and may dictate the precise timing of cell death [3]. Yet other cellular pathways, such as those activated by the tumor suppressor gene, p53, connect apoptotic mechanisms with extrinsic and intrinsic signals [4], [5], [6] that also control cell death [7].

The process of apoptosis has been extensively studied using a wide array of technologies such as microarrays, whole genome small interfering RNA (siRNA) screens and proteomics. This provided valuable insights into genes and proteins that regulate apoptosis [8], [9], [10], [11]. For example, microarray analyses of cells undergoing p53-induced apoptosis suggested that expression of the Apaf-1 gene as well as other proapoptotic genes induced by p53 directed cells into apoptosis [9]. Use of microRNAs and siRNAs identified genes such as *CDK4* that modulates TRAIL-induced apoptosis [11]. Other small interfering RNA screens highlighted the importance of *E2F1-PUMA* and *ERK-MAP3K8* signaling in thapsigargin-induced apoptosis [8]. These studies emphasized the remarkable diversity of signaling pathways that triggered apoptosis in response to distinct noxious stimuli.

Fragmentation into a characteristic DNA “ladder” with fragments representing multiples of approximately 180 bp was proposed to result from intranucleosomal cleavage of genomic DNA during apoptosis [12]. Genomic DNA fragmentation is also frequently monitored by the terminal deoxynucleotidyl transferase-mediated nick end labeling (TUNEL) using biotinylated dUTP [13] but the precise sequences cleaved and the DNases responsible are still largely unknown. For example, apoptosis-inducing factor (AIF) promotes the degradation of chromatin into high molecular weight fragments of approximately 50 kilobases [14]. This pattern may reflect the higher ordered organization of the genomic DNA into complex 3-dimensional structures mediated by chromatin interactions. Yet other factors, such as DNase II, are present in the neighboring cells and can further degrade engulfed or phagocytosed DNA in the final pattern of DNA fragments seen during apoptosis [15].

Growing evidence points to caspase-activated deoxyribonuclease (CAD), also known as the DNase Fragmentation Factor (DFF), as the primary DNase that cleaves genomic DNA within the dying cells into the characteristic pattern or nucleosomal “ladder” seen during apoptosis [16]. As noted above for apoptosis, CAD plays roles outside human disease, by promoting cell differentiation through the induction of DNA breaks [17]. Interestingly, CAD knockout mice show an increased susceptibility to cancer [18], and CAD mutations are also commonly seen in human cancers [19], [20]. Also, CAD is aberrantly expressed in some cancer cells. In human hepatoma cells, alternatively spliced CAD transcripts have been found, potentially contributing to the ability of these cells to circumvent apoptotic signaling that leads to cell death [21]. These data not only suggest a close link between CAD-mediated DNA cleavage and cancer but when combined with the observed resistance of most human cancer cells to apoptosis [22], they hint at the aberrant activation of CAD and consequent DNA breaks in gene translocations and other genomic perturbations in the process of cell transformation and oncogenesis.

Previous experiments suggested that genomic DNA is degraded in a “homogenous” manner during apoptosis [23]. However, these and other studies of apoptotic DNA were limited in their throughput, and yielded conflicting results on whether apoptotic breakpoints were biased towards gene-poor (heterochromatin) or gene-rich (euchromatin) regions of the chromatin. For example, fluorescence *in situ* hybridization experiments showed a distinct bias in the apoptotic breakpoints towards heterochromatin [24]. By contrast, Sanger sequencing of cloned apoptotic DNA fragments in chicken liver suggested that apoptotic DNA cleavage sites were non-random and significantly biased towards genes [25]. Ligation-mediated PCR of selected cleavage sites also suggested a non-random mechanism for DNA fragmentation during apoptosis [26].

One of the implications of selective cleavage of genomic DNA during apoptosis is that the free or exposed DNA ends may be more prone to conferring gene translocation if rapid DNA repair occurred and the cell escaped apoptosis through acquiring a growth advantage, or if incorporated into the genome of a neighboring cell.

Rapid DNA repair upon a catastrophic genome shattering event which would otherwise lead to apoptosis of the cells has been suggested to occur in cancer cells, in a process called “chromothripsis”, whereby up to hundreds of genomic translocations may occur in a single cellular event [27]. While chromothripsis has been suggested to occur because of shattering of a condensed chromosome during mitosis by ionizing radiation, another mechanism for chromothripsis may be that apoptosis leads to widespread genomic fragmentation, which may then be repaired in the cell, and the resulting rearrangements can lead to a growth advantage.

As an alternative mechanism, fragmented apoptotic DNA from tumors was shown to undergo “horizontal gene transfer”, namely the ability to transfer into the genome of surrounding cells, most likely through their engulfment of apoptotic or dying cells [15]. This process can be remarkably efficient [28], requires CAD [29], and may induce tumor formation *in vivo*, possibly through the induction of gene translocations [30]. The tumor suppressor gene p53 prevents cells from replicating the transferred DNA, providing a possible explanation for the ability of cells carrying a mutant p53 gene to acquire genetic alterations [15]. Furthermore, studies of the *MLL* gene suggested that selected apoptotic DNA breakpoints closely mapped to sites of translocations in this gene noted in human cancers [31], [32], [33], [34], [35]. We speculate that integration of apoptotic DNA into the genomes of normal cells through horizontal gene transfer may lead to observed “chromothripsis” events.

**Figure 1:**
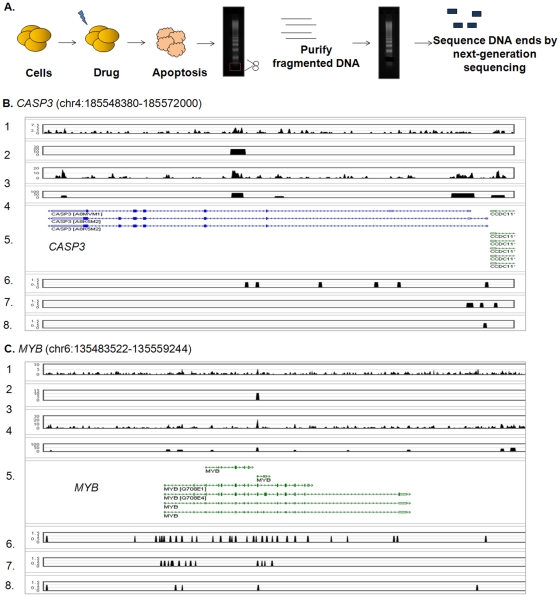
The Apoptoseq Method. A. Schematic of the Apoptoseq methodology is shown. B. Screenshot of the apoptotic DNA patterns for the *CASP3* gene Track labels: 1 – AHH001 biological replicate 1 Apoptoseq sequence density. 2 – Peaks called from AHH001. 3 - AHH002 biological replicate 2 Apoptoseq sequence density. 4 – Peaks called from AHH002. 5 – UCSC Genes. 6 – DNase I hypersensitivity Sequencing Peaks (1 indicates a peak is present, 0 indicates no peak is present) [53]. 7 – H3K4me3 ChIP-Seq Peaks (1 indicates a peak is present, 0 indicates no peak is present) [53]. 8 – CTCF ChIP-Seq Peaks (1 indicates a peak is present, 0 indicates no peak is present) [53]. C. Screenshot of the apoptotic DNA patterns for the *MYB* gene. Track labels follow labels in Figure 1B.

To better understand the relationship between apoptosis, DNA breakpoints and gene translocations, a genome-wide map of apoptotic DNA breakpoints is required. To analyze DNA breakpoints in a high-throughput, *de novo* and global manner, we developed a novel “Apoptoseq” methodology (Figure 1, panel A) described here. The application of the “Apoptoseq” methodology to Actinomycin D-treated HL-60 human leukemic cells yielded the first full genome apoptotic DNA breakpoint map (Figure S1; Table 1; Table S1). These data established that apoptotic DNA breakpoints were non-random, and frequently associated with actively transcribed genes residing in open regions of the chromatin and at sites where transcription factors are known to bind. Comparison of the apoptotic cleavage sites with sites of frequent translocations found in human cancers suggests a possible link between the cellular mechanisms that contribute to apoptosis and gene translocation.

**Table 1:** Statistical Analyses of Apoptoseq Libraries.

Sample	Total Reads	Uniquely mapped	Peaks
Actinomycin D-treated HL-60 cells, Biological Replicate 1 **(AHH001)**	38 million	15 million	7,413
Actinomycin D-treated HL-60 cells, Biological Replicate 2 **(AHH002)**	108 million	38 million	255,488
Composed of 2 technical replicates (AHH002A, AHH002B)			
**AHH002A**	55 million	18 million	132,580
**AHH002B**	54 million	20 million	125,774

Apoptoseq libraries were generated from Actinomycin D-treated HL-60 cells and analyzed as described in Methods.

## Results and Discussion

### Apoptoseq

During apoptosis, cellular DNases cut internucleosomal regions into double-stranded DNA fragments of 180–200 bp [12], which are seen as a “ladder” of DNA fragments following agarose gel electrophoresis. For “Apoptoseq”, we excised the most highly cleaved 180 bp DNA band, on the basis that this represents the most cleaved fraction of the apoptotic ladder, and is therefore a good candidate for initial studies of the apoptotic ladder. In later experiments, other higher migrating DNA bands were also analyzed. As the apoptotic DNA possessed either blunt ends or 3′ overhangs [36], the DNA fragments were subjected to blunt-ending, followed by sequencing adapter ligation as described in Methods. The ensuing DNA sequencing protocol utilized the procedure for the ABI SOLiD [37] next-generation sequencing library construction. The fact that apoptotic DNA breaks could be ligated was previously established using ligation-mediated PCR to amplify these apoptotic DNA fragments [26]. We sequenced the equivalent of 1–2 octets of one plate of ABI SOLiD per library, for all libraries, and 50 base tags were produced (Figure 1, panel A).

We then aligned raw sequences to the human genome to identify the origins of the DNA fragments. Specifically, we mapped all reads to the hg19 human genome assembly using Batman v2.0 (Tennakoon *et al*, manuscript in preparation). We sequenced the ends of the 180 bp DNA fragments, in numerous repeat experiments. We predicted that randomly cleaved DNA would be less likely to be sequenced in many repeat experiments and thus, would distribute in a scattered or random manner across the genome. In contrast, the apoptotic cleaved DNA would be more clustered. Specifically, we used a Chromatin Immunoprecipitation with Sequencing (ChIP-Seq) software application, MACS [38], to call the peaks for these reads. We adopted the MACS software for identifying peaks in the full-genome apoptotic maps, as the 180 bp fragments enriched at apoptotic breakpoint cut sites were analogous to transcription factor binding sites enriched in 180 bp DNA fragments using ChIP-Seq methodology. Thus, an Apoptoseq peak represents a region of non-random clustered tags from the ends of DNA cut during apoptosis and a putative apoptotic breakpoint.

### Identification of non-random apoptotic breakpoints

We analyzed two biological replicates (libraries AHH001 and AHH002) of HL-60 cells treated with Actinomycin D for 19 hours (Table 1). Actinomycin D, which has been used to treat cancers, such as gestational trophoblastic neoplasia [39], induces apoptosis through the inhibition of RNA polymerase [40].

After removing duplicated reads arising from clonal PCR amplification and performing peak-calling to identify statistically significant sequence clusters (or peaks), we noted 7,413 peaks in one – AHH001 - library and 255,488 peaks in the other – AHH002 - library (Table 1; Table S1; Table S2). These data indicated that at least a fraction of apoptotic breakpoints were non-random as sequencing random fragments would not be expected to yield statistically significant peaks. The peaks showed a wide distribution in intensities. In general, there were fewer high-intensity peaks and many more low-intensity peaks (Figure S2).

After accounting for sequencing depth, there was still a significant difference in the numbers of peaks from the two libraries. To investigate whether these represented technical variability in the preparation of the libraries, we undertook two technical replicates of one of the biological replicates (AHH002A and AHH002B). The technical replicates were prepared from the same sample. Therefore, the comparison of these two replicates served as an important control to evaluate potential variations in the library preparation. Here, we found good agreement between these technical replicates, which were sequenced to similar sequencing depths, with 132,580 peaks obtained for AHH002A and 125,774 peaks for AHH002B (Table 1). Moreover, upon closer inspection of a number of cleavage sites, we found that the technical replicates displayed remarkably similar patterns (Figure S3, panel A). Overlapping the peaks, we found a high degree of similarity with 58% of AHH002A sites overlapping with those in AHH002B (51584 of 125774) (Figure S3, panel B). The intensities of peaks in the two technical replicates also showed a high degree of reproducibility (r = 0.837; Figure S3, panel C). Based on these findings, we attributed the differences in peaks in the two biological replicate libraries to be due to biological and not technical variability.

**Figure 2:**
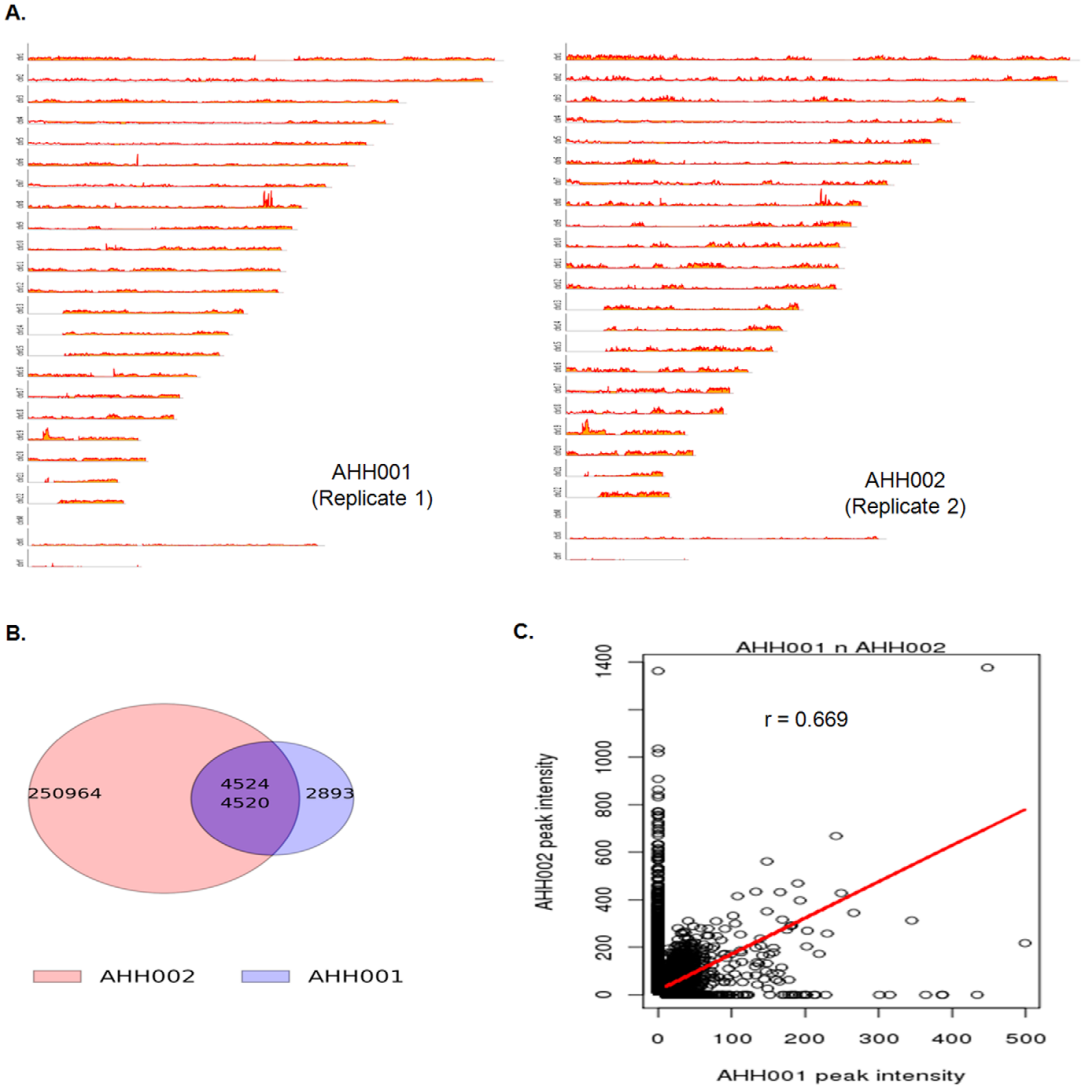
Comparison of Apoptoseq Replicates. A. Genome-wide graphs of AHH001 and AHH002. B. Venn diagram of overlaps between AHH001 and AHH002. The number in the pink circle indicates AHH002 peaks that did not overlap with AHH001 peaks. The number in the blue circle indicates unique AHH001 peaks that did not overlap with AHH002 peaks. The top number in the purple or overlapping region indicates AHH002 peaks that overlap with AHH001 peaks and the bottom number indicates AHH001 peaks that overlapped with AHH002 peaks. C. Graph comparing the peak intensities of overlapping regions in AHH001 and AHH002.

Further examination of the biological replicates, AHH001 and AHH002, focused on specific cleavage sites (Figure 1, panels B & C, Figure S4, Panel A to F). We found good agreement in the location of the peaks in the two libraries (Figure 1, panels B & C: Figure S4, panels A to F) with certain sites showing similar trends between both replicates. However, the signal-to-noise ratio in only one biological replicate was large enough to clearly call an apoptotic breakpoint peak (Figure S4, panel B). Plotting the data as genome-wide maps revealed peaks in all chromosomes (Figure 2, panel A), indicating that Apoptoseq methodology identified apoptotic breakpoints in a global manner. The genome-wide maps from the two biological replicates were also very similar. The majority of peaks in AHH001 (4520 of a total of 7413 sites, 61%) overlapped with those in AHH002, establishing the reproducibility of Apoptoseq (Figure 2, panel B). Comparison of the peak intensities of AHH001 with AHH002 indicated a correlation (r = 0.669; Figure 2, panel C), further emphasizing the reproducibility of the Apoptoseq method. Possible reasons for a lack of overlap between the two biological replicates may include variation in the time taken to respond to the apoptotic stimulus, such that cells in later stages of apoptosis experienced greater DNA fragmentation than cells at earlier stages of apoptosis. Alternatively, sample-to-sample variation in chromosomal regions susceptible to CAD cleavage could contribute to this outcome. Further work will be clearly needed to evaluate the basis for the biological variation.

**Figure 3:**
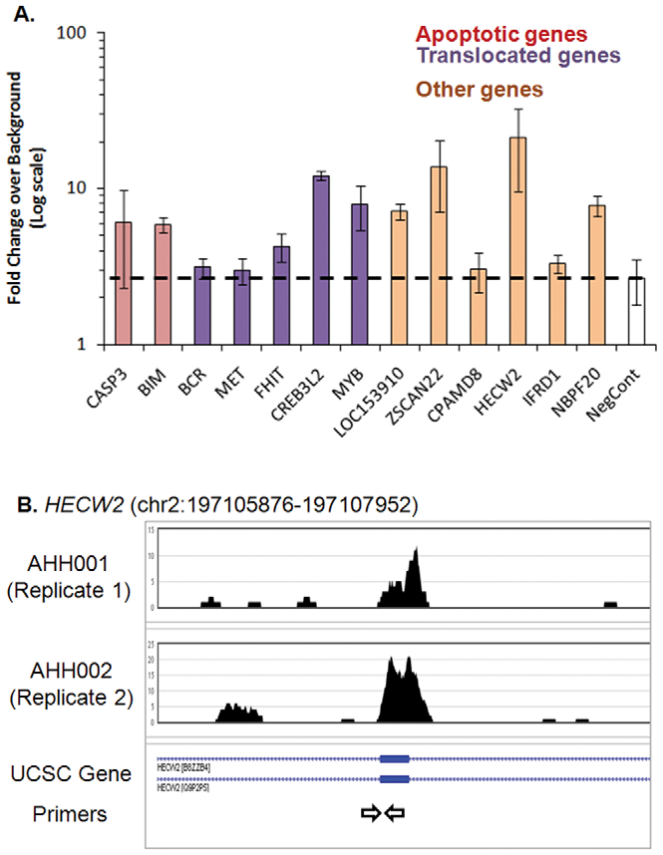
Apoptoseq Validation. A. Quantitative PCR validations were performed using primers against apoptotic peaks, with normalization against a pair of negative control primers that did not map to apoptotic peaks. In addition, the 180 bp apoptotic DNA was normalized against uncut genomic DNA. A second pair of negative control primers “NegCont” was used to set the threshold for calling sites that passed. From this analysis, all the sites passed. The columns represent the average of three replicates. Error bars indicate standard errors. B. Screenshot showing the apoptotic peak at a region that was validated, *HECW2*.

We validated 15 distinct loci for apoptotic cleavage including 13 found in both AHH001 and AHH002 libraries and 2 negative controls quantitative PCR (Figure 3, panels A & B; primers listed in Table S3). These loci were chosen from breakpoints near apoptotic genes (e.g. *CASP3*) and frequently translocated genes (e.g. *MYB*), as well as regions of the genome without any known relation to either apoptosis or translocations. The loci are identified by their position with respect to the closest genes. We also used quantitative PCR to analyze the 180 bp band that was gel-excised from apoptotic ladders prepared in the lab. Apoptosis was confirmed by western immunoblot analysis of cleaved PARP (Figure S5, panel A). We normalized the data against uncut, purified HL-60 genomic DNA. This took into account any chromosomal abnormalities in HL-60 cells. To adjust for any differences in loading, we normalized our data against one negative or control primer (Table S3) complementary to genomic DNA regions that were not subject to apoptotic cleavage. Again, some variability between biological replicates was seen. While 4 of the 13 primer pairs (*CPAMD8*, *BCR*, *IFRD1*, *MET*) showed modest enrichments as compared with the negative control, 9 primer pairs showed excellent enrichments against the same control. Moreover, 3 primer pairs (*ZSCAN22*, *HECW2*, *CREB3L2*) showed remarkably high enrichments (Figure 3, panel A). We also ran qPCR reactions with 7 primer pairs against 180 bp fragments from commercially available DNA derived from Actinomycin D-treated HL-60 cells and observed similar enrichments to that from apoptotic DNA prepared in-house (Figure S5, panels B & C).

Next, we examined apoptotic breakpoints in larger DNA fragments in the apoptotic ladder. Specifically, we analyzed the 360 bp DNA fragments from Actinomycin D-treated using 7 primer pairs in qPCR (Figure S6, panel A). The 360 bp fragments showed similar general trends in terms of the genes cleaved as the 180 bp fragments (Figure S5, panel B), but showed slightly lower fold changes. Interestingly, the *BCR* gene showed a higher fold change in the 360 bp fragments. By contrast, the entire apoptotic ladder showed very low fold changes compared with background (Figure S6, panel B). The latter results are consistent with the notion that large DNA fragments are generated by more limited cleavage by apoptotic DNases and highlight the need to isolate the 180 bp (or 360 bp) fragments to identify the apoptotic breakpoints. While an alternative sequencing methodology could utilize DNA isolation via tags added to the ends of all DNA fragments, validations of this approach would still require quantitative PCR and necessitate isolation of the 180 bp or 360 bp fragments.

Whether apoptotic breakpoints were changed with increasing exposure to cytotoxic drug was assessed by comparing the qPCR results obtained for 180 bp fragments from HL-60 cells treated with Actinomycin D at 4 hours (Figure S6, panel C) and those treated for 19 hours (Figure S5, panel B). However, we observed little difference in the fold inductions of amplified DNA using 7 selected primer pairs. This suggested that DNA fragmentation in the Actinomycin D-treated HL-60 cells had already arrived at an end stage by 4 hours.

### Genes Associated with Apoptotic Breakpoints

**Figure 4:**
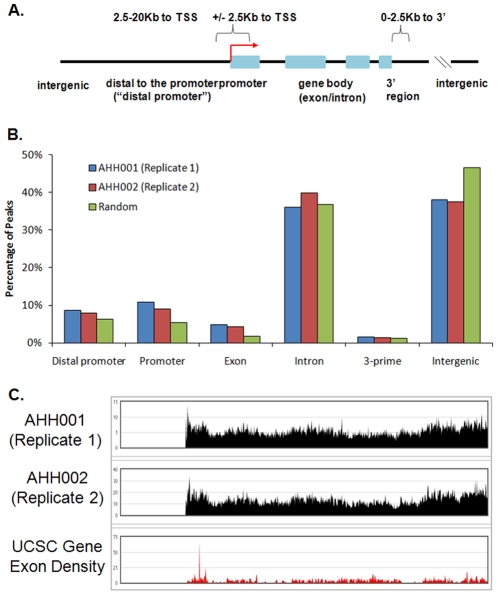
Apoptotic Breakpoints Associated with Genes. A. Diagram of the distinct categories of locations. B. Graph of locations of apoptotic DNA breakpoint peaks. C. Screenshot of apoptotic breakpoint peaks with regards to UCSC Gene exon density in chromosome 14.

The full genome maps of apoptotic breakpoints were compared with UCSC genes for overlaps with putative promoters, exons, introns, 3′ sequences and even some more distal regions from genes (up to 20 kb from the transcription start site) (Figure 4, panel A; Table S2; Table S4). We noted that many apoptotic breakpoints were located within introns, with some present in 5′ and 3′ regions of genes and in exons (Figure S4, panels A to H; Figure 4, panel B). Interestingly, there were significantly more apoptotic breakpoints associated with the regions distal to promoters and in promoter regions as compared to random regions of the genome (Figure 4, panel B; chi-square p-value = 1.06^−11^ for distal promoters in AHH001; chi-square p-value<2.2^−16^ for promoters in AHH001; chi-square p-value<2.2^−16^ for distal promoters in AHH002; chi-square p-value<2.2^−16^ for promoters in AHH002). This data suggested that during apoptosis, intranucleosomal cleavage of genomic DNA was significantly concentrated at the promoters of most likely actively-transcribed genes [41].

In the AHH001 DNA fragment library, 4595 of 7413 breakpoints (62%) were associated with genes. In AHH002, essentially similar (62% or 159611 of 255488) breakpoints were associated with genes. By contrast, only 128255 of 248999 (52%) random sites selected in the whole genome showed an association with genes. In performing the significance calculations, the Pearson's chi-square p-value of AHH001 was 2.5^−201^; and the Pearson's chi-square p-value of AHH002 was 0 (Figure 4, panel B; Table S2; Table S4). This association of apoptotic DNA breakpoints with genes was also seen by comparing gene density with apoptotic peaks, as shown for Chromosome 14 (Figure 4, panel C). As UCSC lists both gene transcripts and genes, both UCSC gene ID (transcript ID) and gene symbol (gene ID) are shown for each breakpoint (Table S2). AHH001 was associated with 6708 gene transcripts comprising of 2807 genes. By comparison, AHH002 was associated with 44179 gene transcripts from 17321 genes (Table S4). Moreover, the GC content of apoptotic breakpoints in AHH001 was 49% while that for AHH002 was 52%, both greater than the average of 41% for the full genome [42]. As genic regions of the genome are associated with higher GC content [43], the observed high GC content of apoptotic breakpoints also suggests a higher gene density at the apoptotic breakpoints.

Interestingly, some genes were highly cleaved during apoptosis, while others were either rarely cleaved or not at all (Figure S4, Table S4). One example of a highly cleaved gene was *FHIT*, a tumor suppressor gene located at a very common fragile site (FRA3B) (Figure S4). As fragile sites are often associated with gene translocations [44], [45], these data suggested that some apoptotic DNA breakpoints might be associated with translocations. Apoptotic breakpoints were also noted in the *MLL* gene in one of the two biological replicates (Figure S4, panel D). *MLL* is a gene, which was previously associated with gene translocation and apoptotic cleavage in human leukemia cells, raising the possibility that other translocated genes may be associated with apoptotic cleavage [31], [32], [33], [34], [35]. Apoptotic breakpoints were also found in the *MYB* gene (Figure S4, panel D), which is commonly dysregulated in human cancers and also found to be susceptible to translocations [46]. Other examples of translocation-prone genes [47] are shown in Figure S4D.

To further explore the link between translocation-prone genes and apoptotic DNA breakpoints, we examined the cancer-associated genes and translocation-associated genes curated by Michael Stratton, Andy Futreal, and colleagues at the Wellcome Trust Sanger Institute [47]. Of the known cancer-associated genes, 70 of 2807 (2.5%; chi-square p-value<0.001) genes were associated with cancers in the AHH001 fragment library, and 308 of 17321 (1.8%; chi-square p-value<0.001) genes in AHH002 were also associated with cancer. This is compared to only 378 of the total of 29209 UCSC genes (1.3%) in the human genome. Of translocation-prone genes, we found that 48 of 2807 (1.7%; chi-square p-value<0.001) genes in AHH001, and 207 of 17321 (1.2%; chi-square p-value<0.001) in AHH002 were associated with translocations, compared with only 252 of the total 29209 genes (0.9%) in the entire human genome (Table S4). While the percentage of apoptotic genes associated with translocations appears low, at least some genes cleaved during apoptosis may result in translocations that are toxic or lethal to cells and thus would not be present in the above cancer database. For example, *CASP3* was cleaved during apoptosis, and this breakpoint was validated by quantitative PCR (Figure 1; Figure 3), but *CASP3* is not associated with any recorded translocations in human cancers.

Interestingly, in addition to *CASP3*, several genes encoding caspases and other factors involved in apoptosis, such as *DFFA* and *DFFB*, both components of the caspase-activated DNase, were also cleaved during apoptosis in at least one replicate (Figure S4, panel E). Thus, it is tempting to speculate that cleavage of proapoptotic genes may attenuate or slow apoptosis, and allow time for cancer cells to incorporate the cleaved DNA fragments into their genome and through the ensuing growth advantages possibly conferred by the ensuing gene translocations to survive and proliferate in the continued presence of the proapoptotic stimuli.

### Further Characterization of Apoptotic DNA Breakpoints

To functionally annotate the DNA breakpoints, we compared a DNase I Hypersensitivity map as an indicator of regions of open chromatin [48], a H3K4me3 ChIP-Seq map as this histone modification is associated with active gene transcription [49], and a CTCF (CCCTC-binding factor) ChIP-Seq map as CTCF is a zinc finger protein which organizes chromatin, acts as an insulator [50], and is also associated with DNase I hypersensitive sites and Sono-Seq open chromatin regions sites [51], [52]. These maps were produced in HL-60 cells by the ENCODE consortium (Stamatoyannopoulos and colleagues, University of Washington) [53].

**Figure 5:**
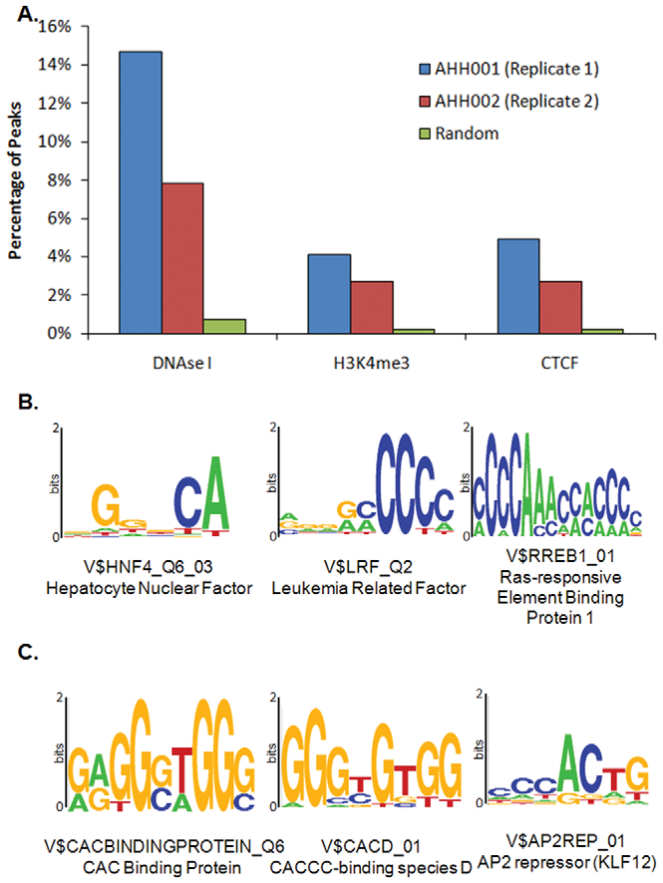
Characterisation of Apoptotic DNA Breakpoints. A. Association of apoptotic breakpoints with H3K4me3 marks, DNase I hypersensitivity marks, and CTCF marks. B. Transcription factor motifs found in apoptotic breakpoints from both AHH001 and AHH002 biological replicates. C. Examples of transcription factor motifs found in only one apoptoseq replicate, AHH002.

In contrast to randomly selected sites in the human genome which showed only 0.7% (1823 of 248999) associated with DNase I hypersensitivity, approximately 15% (1090 of 7413) of DNA breakpoints in the AHH001 fragment library and 8% of apoptotic breakpoints in AHH002 (20049 of 255488) were associated with DNase I hypersensitive sites. These associations were significant (Binomial p-value = 0 for both AHH001 and AHH002) (Figure 5, panel A), suggesting that genomic sequences located in regions of open chromatin and actively transcribed were particularly susceptible to cleavage during apoptosis.

In addition, 4% (303 of 7413) of DNA breakpoints in AHH001 and 3% in AHH002 (6922 of 255488) were marked by H3K4me3. By contrast, only 0.2% (598 of 248999) of random genomic sequences were associated with H3K4me3 marks. The association between apoptotic breakpoints and H3K4me3 marks was significant (Binomial p-value = 0 for both AHH001 and AHH002) (Figure 5, panel A), further emphasizing that actively transcribed genes located in regions of open chromatin were particularly prone to apoptotic cleavage.

Finally, 5% (367 of 7413) of DNA breakpoints in AHH001 and 3% in AHH002 (6926 of 255488) were identified as CTCF sites. By contrast, only 0.2% (573 of 248999) of random sites showed CTCF marks. This association (Binomial p-value = 0 for both AHH001 and AHH002) (Figure 5, panel A); pointed to the association of apoptotic DNA breakpoints with insulator regions.

To complete these analyses, we also examined the regions of the DNA breakpoints for the presence of motifs representing potential transcription factor binding sites using the Centdist software [54]. These analyses indicated that the binding sites for selected transcription factors, such as HNF4, LRF, and RREB, were significantly enriched in both fragment libraries (Figure 5, panel B). HNF4 (hepatocyte nuclear factor 4) is part of the hepatocyte nuclear factor/Met axis that is aberrantly expressed in cancers [55]. Moreover, expression profiling has revealed that HNF4 target genes were often associated with apoptosis [56]. LRF, also called Pokemon, is another transcriptional repressor associated with cancer [57]. In contrast to LRF, which is a transcriptional repressor, the transcription factor, RREB (*Ras*-responsive element binding protein), which activates p53 to control apoptosis [58], was also identified in these analyses. Interestingly, two *RAS* genes (*H-RAS* and *K-RAS*) also displayed significant apoptotic breakpoints in at least one biological replicate (Figure S4, panel F), potentially suggesting that cleavage of some cancer-associated genes may also occur during apoptosis. Interestingly, many of these transcription factors bound poly-C motifs, which was consistent the high GC content of the apoptotic DNA breakpoint peaks highlighted in this study. Specifically, we found transcription factors, such as AP-2rep (KLF12), from the cancer-associated Krüppel-Like Factor (KLF) family, which bind a CACCC motif (Figure 5, panel C) [59].

In conclusion, many human cancers have been separately linked to gene translocations and altered control of apoptosis. Studies of apoptosis using microarrays, proteomics, and other technologies are limited to the analysis of transcribed genes and/or their protein products. While valuable in providing insights into the apoptotic process, they have not revealed the role of DNA strand breaks that occur during apoptosis.

In this work, we developed a new method, Apoptoseq, to investigate the specific regions of the genome cleaved during apoptosis. We successfully applied Apoptoseq to study end-repaired fragments of apoptotic DNA, suggesting that even clinical cancer samples which show high amounts of fragmented apoptotic DNA may still be amenable to next-generation sequencing.

Our results illustrated a pattern of non-random DNA breakpoints in apoptotic cells whereby specific DNA regions rather than sequences experiences preferential cleavage by CAD, which activated during apoptosis. The selectivity of this DNA cleavage mechanism may spare certain DNA elements and genes, which can be engulfed by other cells to promote horizontal gene transfer, and the incorporation and activation of these genes may contribute to cancer progression. The cleavage of some genes such as *CASP3* may preclude the activity of these genes following their engulfment. Alternatively, the cells may be able to escape apoptosis. DNA repair of the apoptotic DNA fragments may generate gene translocations, which in turn may allow cells that to gain some growth advantage and progress into tumors. As many translocations arise in non-random “hotspots” [60], and since “chromothripsis” (a catastrophic genome shattering event similar to that of the induction of apoptotic DNA cleavage [27]) has been described to occur in cancer cells, we speculate a potential mechanistic link between DNA cleavage during apoptosis and gene translocations (Figure S7).

In *vitro* studies have failed to identify a unique DNA sequence motif recognized by CAD [61]. In this regard, our findings suggest that gene-rich regions in open regions of chromatin bound by specific transcription factors and actively transcribed may be particularly susceptible to cleavage during apoptosis suggesting a more complex mode of recognition of the genome by CAD. As many histones may be dephosphorylated upon apoptosis, this and other epigenetic mechanisms may also induce chromatin conformations that facilitate the access to CAD [62]. Currently, the impact of distinct apoptotic signaling pathways or mechanisms in the pattern of DNA cleavage remains unknown. Apoptoseq analyses of normal and cancer cells exposed to different cytotoxic agents could provide new insights into the diversity of DNA breakpoints elicited by anticancer drugs and thereby point their usefulness as genetic fingerprints or biomarkers of drug-induced cell death. In particular, future studies using non-genotoxic inducers of apoptosis, such as corticosteroids, may be very useful in potentially differentiating DNA breaks induced by a genotoxic agent, as used in this study. Apoptotic biomarkers might be obtained that highlight when the activation of apoptosis may facilitate gene translocations to promote drug resistance and be a prelude to the formation of more aggressive tumors.

## Materials and Methods

### Analysis of Apoptosis

Apoptosis was confirmed by Western Blot analyses (three replicates) as previously described [63] using cell lysates prepared in ice-cold RIPA buffer supplemented with Protease Inhibitor Complete cocktail (Roche). Antibodies that recognized cleaved PARP (Abcam), as well as anti- tubulin antibodies (Sigma) were used for immunoblotting and detected using with horseradish peroxidase-linked goat anti-mouse IgG and goat anti-rabbit IgG from Santa Cruz Biotechnology.

### Isolation of Apoptotic DNA

Apoptotic DNA ladders generated by treating HL-60 cells with 0.5 µg/ml Actinomycin D (Sigma) for 19 h were prepared using the Merck Suicide-Track kit. Selected DNA fragments were subjected to RNase ONE (Promega) digestion followed by Proteinase K treatment (Sigma). The 180 bp or 360 bp bands were excised following electrophoresis on 1.9% agarose gels and further purified using Qiaquick gel extraction kit (Qiagen). The purified DNA was quantitated by Nanodrop and three replicates prepared for analysis by quantitative PCR.

### Preparation of Genomic DNA

As a control for qPCR analyses and normalizing for inherent genomic aberrations, HL-60 cells (from Marc Fivaz, Duke-NUS Graduate Medical School) were grown in RPMI-1640 media supplemented with 10% heat-inactivated Fetal Bovine Serum at 37°C in a humidified 5% CO_2_ incubator. RNA-free genomic DNA was harvested from the cells using a Sigma genomic DNA extraction kit according to the manufacturer's protocols.

### Generation of Apoptoseq Libraries

Apoptotic DNA was obtained from lyophilized 1×10^6^ HL-60 human leukemia cells treated with 0.5 µg/ml Actinomycin D for 19 h provided with the Merck Suicide-Track kit (positive control part no: D00073752). An additional biological replicate was also obtained from Merck (positive control part no: D00063279). The 180 base-pair band was excised from 1.9% agarose gels and purified with a Qiaquick gel extraction kit (Qiagen). The smallest DNA fragments were used for Next-Gen sequencing as this represented the most extensively cleaved apoptotic DNA fragments that would likely encompass, both early and late DNA breaks. An added benefit is that 180 bp DNA fragments are well suited to next-generation sequencing although other bands could also be examined in future experiments. Representative agarose gel images are shown in Figure S1. Next, we subjected the DNA preparation to RNase ONE (Promega) digestion to remove any contaminating RNA and the resulting DNA was purified using the MinElute kit (Qiagen) to remove the RNase.

The concentration of the purified 180 bp DNA was quantitated using Nanodrop, visualized by Agilent Bioanalyzer to ensure that the pure DNA was submitted to 1^st^ BASE Pte Ltd for next-generation sequencing ABI SOLiD library preparation and 50-base single-end sequencing according to manufacturer's protocols for the ABI SOLiD 3 system. The equivalent of one octet (one-eighth of a SOLiD 3 sequencing plate, onto which DNA is placed for sequencing) was used per library for one replicate of Actinomycin D-treated HL-60 cells (replicate no: D00073752). This library was labeled AHH001 (Table 1).

To investigate the possibility of large variations in library construction that could subsequently confound the sequencing, we prepared two technical replicate libraries. The two technical replicate libraries were prepared from replicate no: D00063279 and each sequenced with the equivalent of one octet. This library was labeled AHH002, and the two technical replicate libraries were identified as AHH002A and AHH002B (Table 1).

The term “equivalent” is used to only indicate that barcoding of samples and pooled sequencing was performed on samples followed by deconvoluting the barcodes. Barcoding and pooled sequencing was performed in order to reduce sequencing costs, by allowing for different samples to be sequenced together in the same DNA plate.

### Identification of DNA Breakpoints by Apoptoseq

First, we aligned raw DNA sequences to the human genome to identify the origins of the DNA. Specifically, ABI SOLiD data was mapped to the human genome assembly hg19, using Batman (http://code.google.com/p/batman-aligner/, Tennakoon *et al*, manuscript in preparation). Batman is a Burrows-Wheeler-Transform-based (BWT) method that quickly maps short sequences to a reference genome. We sequenced the ends of the 180 bp DNA fragments, in numerous repeat experiments. We predicted that randomly cleaved DNA would be less likely to be sequenced in many repeat experiments and thus, would distribute in a scattered or random manner across the genome. In contrast, the apoptotic cleaved DNA would be more clustered. Specifically, we performed “peak-calling” (“peaks” indicate clusters of regions which were sequenced frequently) on the uniquely mapped reads with identical duplicates (which are likely the result of clonal PCR amplifications) merged using the MACS package frequently used for ChIP-Seq applications [38]. Visual inspection of the data and preparation of screenshots was carried out through uploading tracks of the results to an in-house BASIC browser (Mulawadi et al., manuscript in preparation). Peaks within highly repetitive satellite regions as indicated by RepeatMasker (Smit, AFA, Hubley, R & Green, P. RepeatMasker Open-3.0.1996–2010 Program; http://www.repeatmasker.org) were attributed to noise due to non-specific mapping and excluded from further analyses, as were peaks within ChrY, noise arising from HL-60, a female cell line. Peaks present in known copy number changes in HL-60 cells [64] were not excluded from these analyses, but were flagged in Table S2.

### Analyses of Apoptotic DNA Breakpoints

To calculate overlaps between the two libraries, first, we standardized Apoptoseq peaks through calculating the centre of each peak +/−200 bp. Next, we identified all overlaps, requiring at least 1 bp to call an overlap. We then listed this region as an “overlapping” apoptotic breakpoint in library A. To calculate the percentage of overlaps for library A, we took the number of overlapping apoptotic breakpoints divided by the total number of apoptotic breakpoints for the library A.

Gene analysis was undertaken using UCSC gene transcripts [65]. The center of each peak was identified and checked first for overlaps of the peak with gene promoters (+/−2.5 kb around the transcription start sites). If a positive overlap was observed, these peaks were defined as a “promoter peaks”. Next, we checked the remaining peaks for overlaps with exons and where overlapping, these peaks were defined as “exon peaks”. All remaining peaks from above analyses for overlaps with genes were defined as “intron peaks”. In addition, we checked these peaks for overlaps with regions 2.5 kb downstream of the transcription end sites and where overlapping, defined these as “3′ region peaks”. Peaks overlapping with regions 2.5 kb–20 kb upstream of gene transcription start sites were defined as “distal to the promoter peaks” or “distal promoter”. Peaks, classified as “intergenic”, showed no association with any genes.

We analyzed all apoptotic DNA breakpoint peaks for percentage GC content. H3K4me ChIP-Seq, CTCF ChIP-Seq and DNaseDNase I hypersensitivity maps produced from HL-60 cells by Stamatoyannopoulos and colleagues at University of Washington as part of the ENCODE consortium [53] were downloaded from the UCSC Genome Browser, and converted from hg18 to hg19 genome assembly using the UCSC LiftOver tool [66], [67]. These peaks were standardized to a range of 149 bp. If the center of an Apoptoseq peaks fell within the H3K4me3, CTCF, and DNase I hypersensitivity peaks, this identified the overlapping peaks.

To analyze the associations between apoptotic breakpoint-associated genes, cancer-associated genes, and translocation-associated genes, we utilized the list of UCSC genes, where genes encoding distinct isoforms of the same gene product were counted as one gene. Through this, we obtained a list of 29,209 genes within the entire human genome, which were analyzed for overlaps with the cancer-associated and translocation-associated genes curated by Wellcome Trust Sanger Institute [47].

For the random analyses, 250,000 peaks were simulated from the human genome. After removal of peaks within chromosome Y and satellite regions, 248,999 peaks were examined for their distributions with respect to genes, H3K4me3 data, CTCF data and DNase I hypersensitivity.

### Motif analyses

In addition, we performed transcription factor binding site motif analyses on the full dataset of peaks using Centdist [54].

### Statistical analyses

We used Pearson's chi-square statistical tests to calculate the significance of the association between apoptotic DNA breakpoints and genes, including the translocated genes. We used the binomial distribution to calculate the significance of the association between apoptotic DNA breakpoints and H3K4me3 ChIP-Seq, CTCF ChIP-Seq or DNaseDNase I hypersensitivity peaks.

### Quantitative PCR

Quantitative PCR was performed on 180 bp or 360 bp apoptotic DNA excised from agarose gels, or the entire apoptotic DNA ladder (all apoptotic DNA purified from the Merck Suicide track kit, without further purification by agarose gel electrophoresis) from Actinomycin D-treated HL-60 cells with apoptotic site-specific primers (Table S3), with double normalization against uncut genomic DNA (prepared by a Sigma genomic DNA kit according to manufacturer's protocols, including a RNase A-treatment) at the same site and against a negative control primer (which hybridized to a region without high apoptosis cleavage as previously used [68]). The delta-delta-Ct Fold change calculation method commonly used in reverse transcriptase quantitative PCR (RT-qPCR) was used for double normalization. 3 biological replicates from the Merck Suicide-Track kit were analyzed (from replicate nos. D00073752, D00063279, D1000431).

The 180 bp or 360 bp purified DNA from apoptotic ladders, or apoptotic ladders, or uncut genomic DNA (1 ng quantitated by Nanodrop or Agilent Bioanalyzer), was used in 10 µl of SsoFast Evagreen reaction mix (Bio-rad) and analyzed on a Biorad iQ5 real-time quantitative PCR machine according to standard SsoFast quantitative PCR programs. At least 3 technical replicates were analyzed per reaction, and the standard error of the technical replicates was established at less than 0.5. Primers were designed by Primer3 [69] or Roche Lightcycler's free quantitative PCR design program (http://www.roche-applied-science.com/lightcycler-online/) at non-repetitive regions identified by Repeatmasker (Smit, AFA, Hubley, R & Green, P. RepeatMasker Open-3.0.1996–2010 Program; http://www.repeatmasker.org) were verified to show sharp melting curves with efficiencies between 90% and 110%, and are listed in Table S2.

## Supporting Information

Figure S1 **Agarose Gel Electorphoresis of Apoptotic DNA.**
(DOC)

Figure S2 **Analysis of Apoptoseq Peaks.**
(DOC)

Figure S3 **Analysis of Technical Replicates.**
(DOC)

Figure S4 **Screenshot examples.**
(DOC)

Figure S5 **Apoptoseq validations.**
(DOC)

Figure S6 **Analyses of apoptotic DNA breakpoints.**
(DOC)

Figure S7 **Hypothesized model for apoptotic DNA fragmentation in cancer development.**
(DOC)

Table S1 **Detailed Statistics.** (Excel Spreadsheet).
(XLS)

Table S2 **List of Apoptotic Breakpoints.** (Excel spreadsheet).
(RAR)

Table S3 **List of primers used.** (Excel spreadsheet).
(XLS)

Table S4 **List of Genes associated with Apoptotic Breakpoints.** (Excel spreadsheet).
(RAR)
