# Supplementary material for: Next-Generation Sequencing of Apoptotic DNA Breakpoints Reveals Association with Actively Transcribed Genes and Gene Translocations
Source: PLoS One. 2011 Nov 8;6(11):e26054. doi: 10.1371/journal.pone.0026054 (PMC3210745; doi:10.1371/journal.pone.0026054)
Supplement: Figure S1 — Agarose Gel Electorphoresis of Apoptotic DNA. (DOC) [file pone.0026054.s001.doc]

**
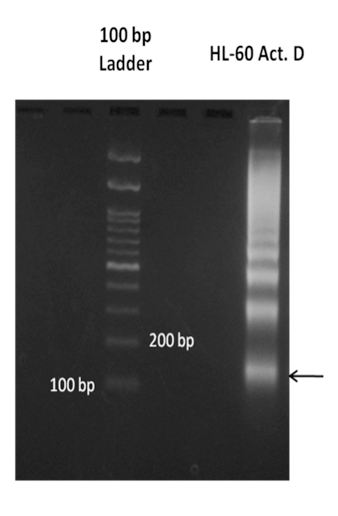
**

**Supplementary Figure 1. Agarose Gel Electorphoresis of Apoptotic DNA.** Representative agarose gel images of apoptotic DNA used to construct Apoptoseq libraries. Apoptotic DNA from Actinomycin D-treated HL-60 human cells was extracted and subjected to electrophoresis on 1.5% agarose gel. The lowest band (approximately 180 bp, indicated by an arrow) was excised and sequenced.
