# Supplementary material for: Next-Generation Sequencing of Apoptotic DNA Breakpoints Reveals Association with Actively Transcribed Genes and Gene Translocations
Source: PLoS One. 2011 Nov 8;6(11):e26054. doi: 10.1371/journal.pone.0026054 (PMC3210745; doi:10.1371/journal.pone.0026054)
Supplement: Figure S2 — Analysis of Apoptoseq Peaks. (DOC) [file pone.0026054.s002.doc]

**
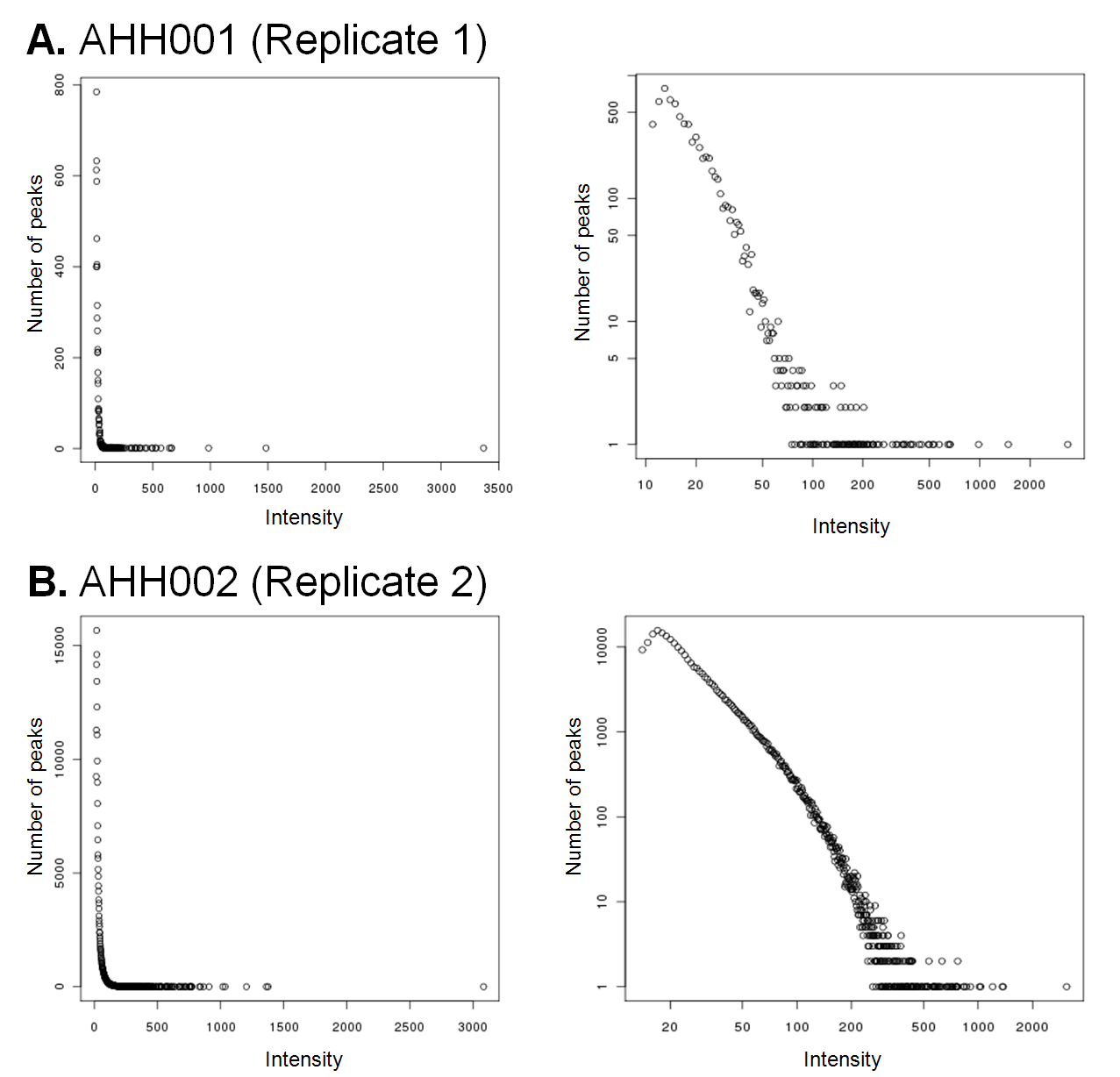
**

**Supplementary Figure 2. Analysis of Apoptoseq Peaks.** A graph of intensities of the peaks against number of the peaks called from the Apoptoseq data shows that there is a wide variation in the levels of apoptotic cleavage at different sites. A. Actinomycin D-treated HL-60 cells replicate 1. B. Actinomycin D-treated HL-60 cells replicate 2.
