# Supplementary material for: Next-Generation Sequencing of Apoptotic DNA Breakpoints Reveals Association with Actively Transcribed Genes and Gene Translocations
Source: PLoS One. 2011 Nov 8;6(11):e26054. doi: 10.1371/journal.pone.0026054 (PMC3210745; doi:10.1371/journal.pone.0026054)
Supplement: Figure S3 — Analysis of Technical Replicates. (DOC) [file pone.0026054.s003.doc]

**
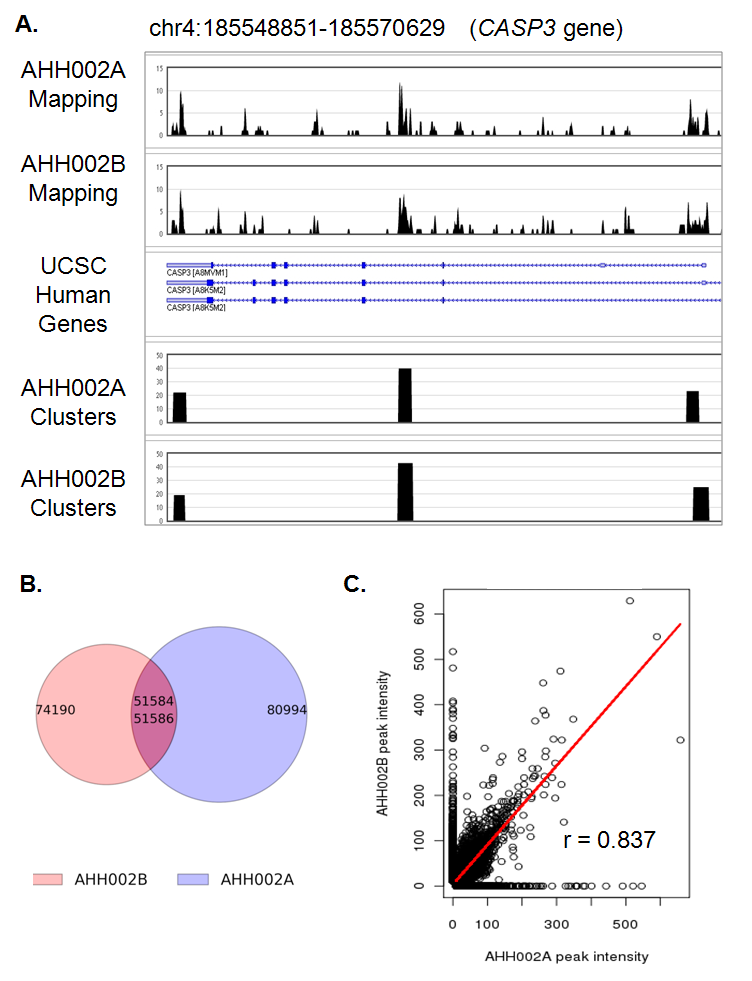
**

**Supplementary Figure 3.** **Analysis of Technical Replicates.** A. An example of AHH002A and AHH002B apoptotic DNA sequence density and designated peaks at *CASP3*. B. Venn diagram of overlaps between AHH002A and AHH002B. The number in the pink circle indicates unique AHH002B peaks while the number in the blue circle indicates unique AHH002A peaks. The top number in the purple segment indicates AHH002B peaks that overlapped with AHH002A peaks and the bottom number indicates AHH002A peaks that overlapped with AHH002B peaks. C. Graph comparing the peak intensities of overlapping regions in AHH002A and AHH002B.
