# Supplementary material for: Next-Generation Sequencing of Apoptotic DNA Breakpoints Reveals Association with Actively Transcribed Genes and Gene Translocations
Source: PLoS One. 2011 Nov 8;6(11):e26054. doi: 10.1371/journal.pone.0026054 (PMC3210745; doi:10.1371/journal.pone.0026054)
Supplement: Figure S4 — Screenshot examples. (DOC) [file pone.0026054.s004.doc]

**
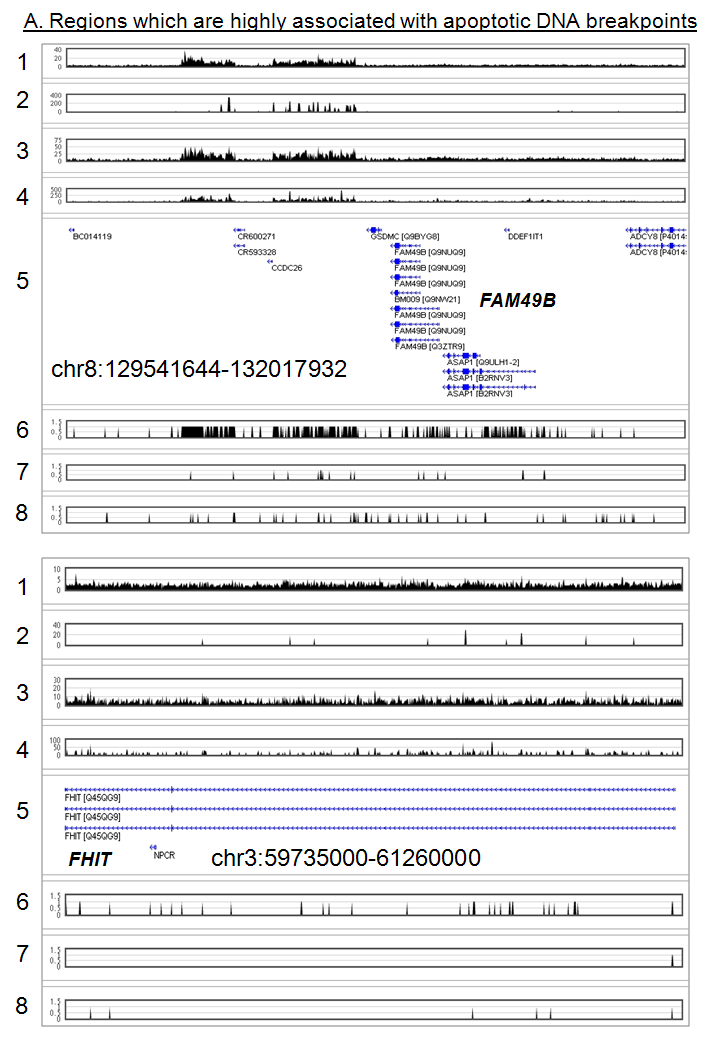
**

**Supplementary Figure 4.** **Screenshot examples**. A. Examples of regions that are highly associated with apoptotic DNA breakpoints. Track labels follow labels in Figure 1B.

**
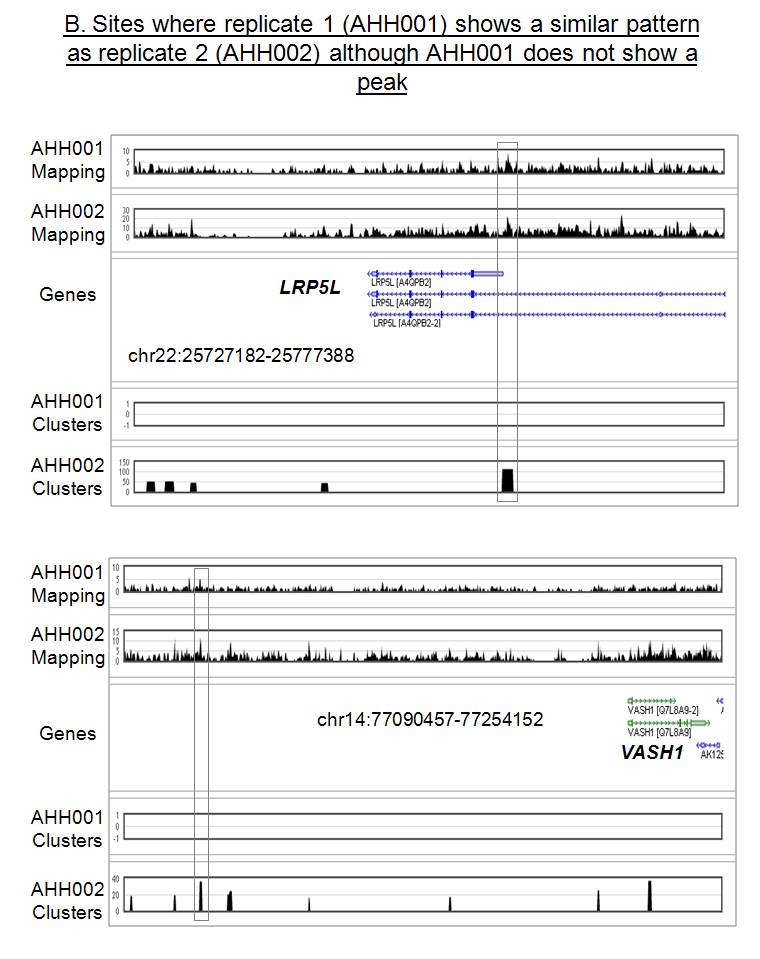
**

**Supplementary Figure 4.** **Screenshot examples**. B. Examples of regions which show similar sequencing density patterns between AHH001 and AHH002 although AHH001 does not show a peak. Track labels follow labels in Figure 1B.

**
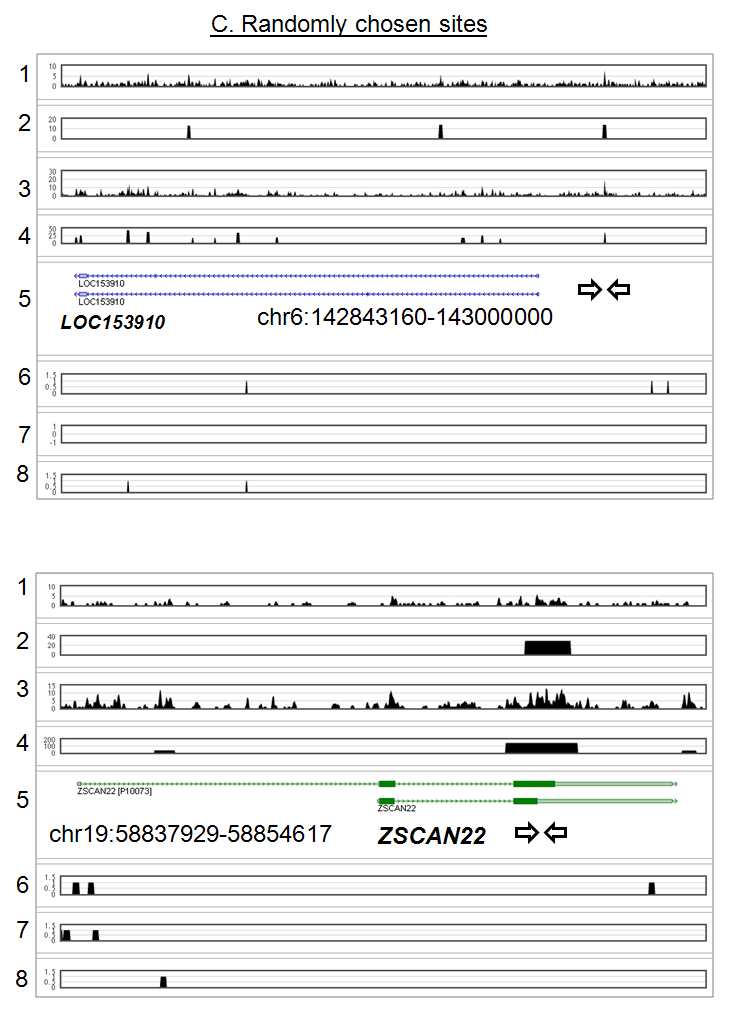
**

**Supplementary Figure 4.** **Screenshot examples**. C. Randomly chosen sites which were used for qPCR validation. Track labels follow labels in Figure 1B. Primer locations are indicated by arrows.

**
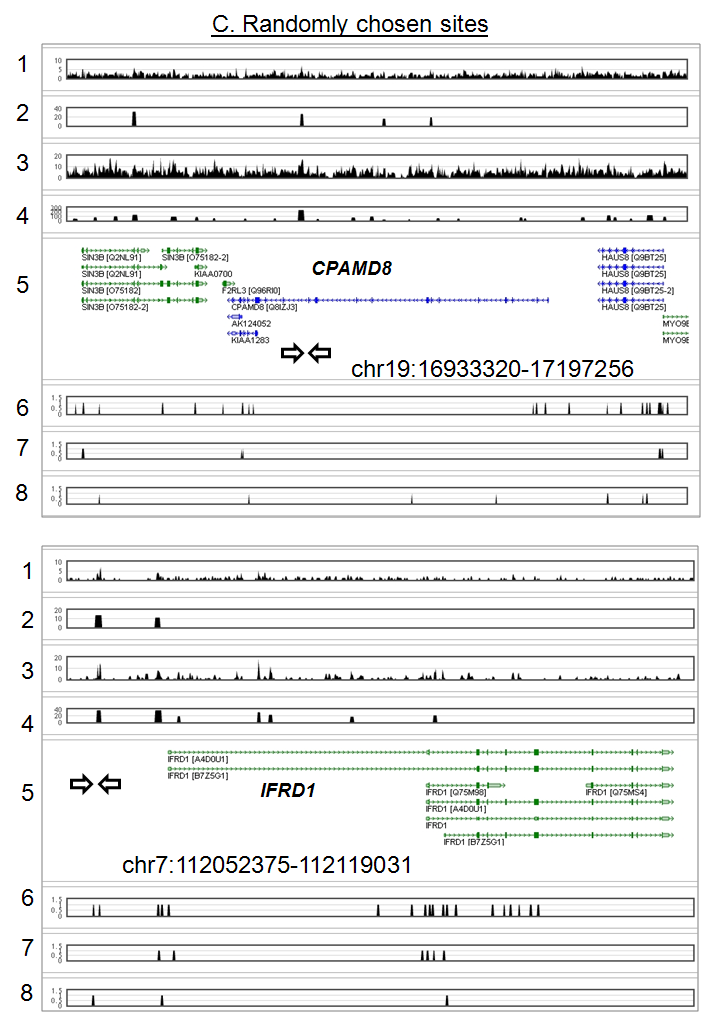
**

**Supplementary Figure 4.** **Screenshot examples**. C. Randomly chosen sites which were used for qPCR validation. Track labels follow labels in Figure 1B. Primer locations are indicated by arrows. (Continued from previous page).

**
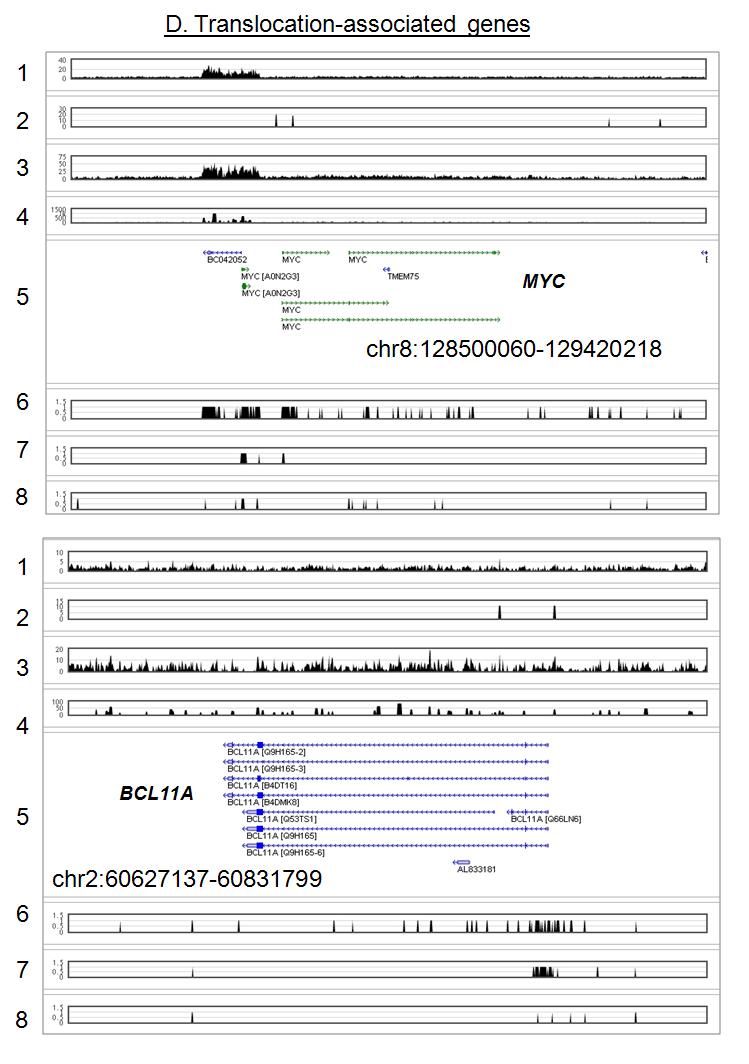
**

**Supplementary Figure 4.** **Screenshot examples**. D. Examples of regions associated with translocations. Track labels follow labels in Figure 1B.


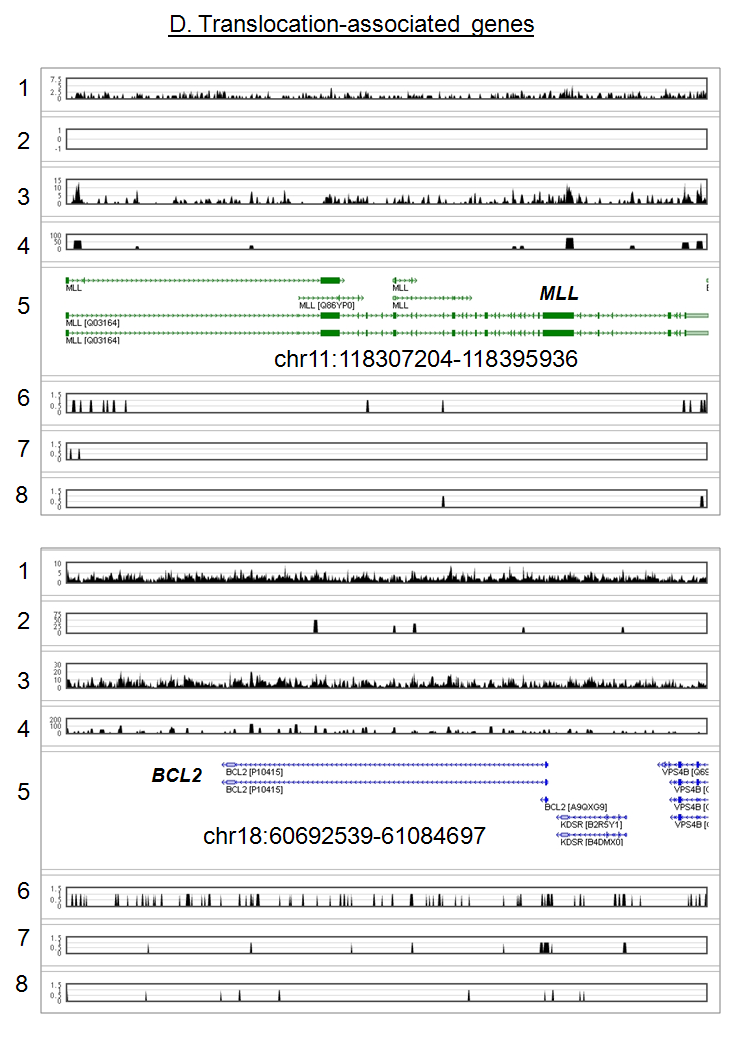


**Supplementary Figure 4.** **Screenshot examples**. D. Examples of regions associated with translocations. Track labels follow labels in Figure 1B. (Continued from previous page).

**
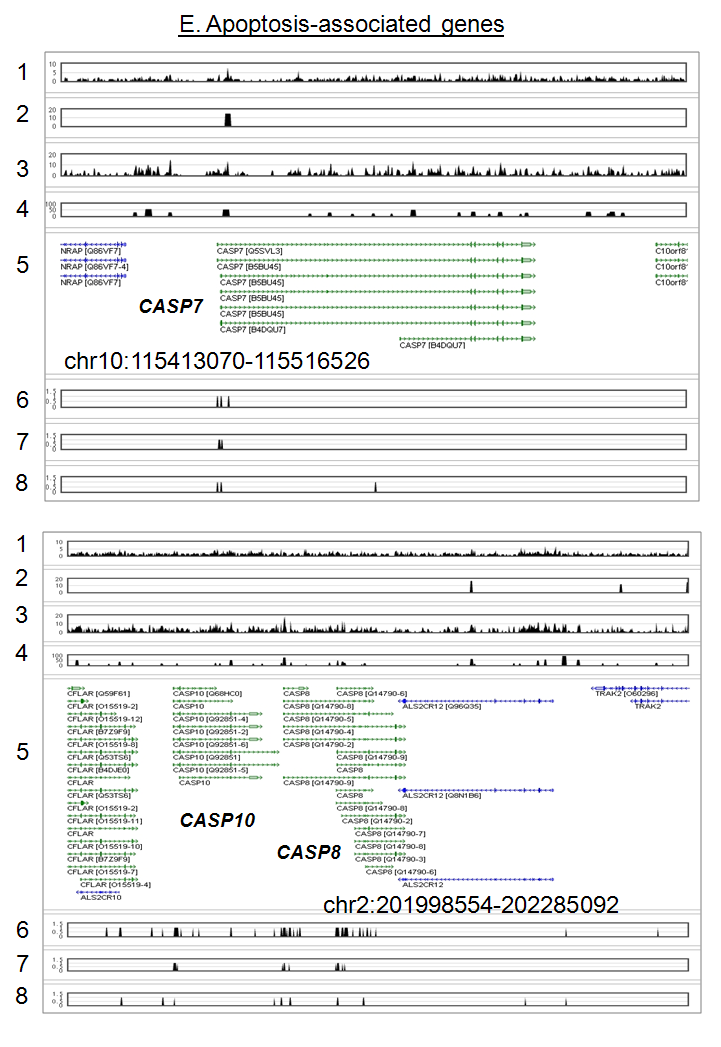
**

**Supplementary Figure 4.** **Screenshot examples**. E. Examples of regions with apoptosis-associated genes. Track labels follow labels in Figure 1B.

**
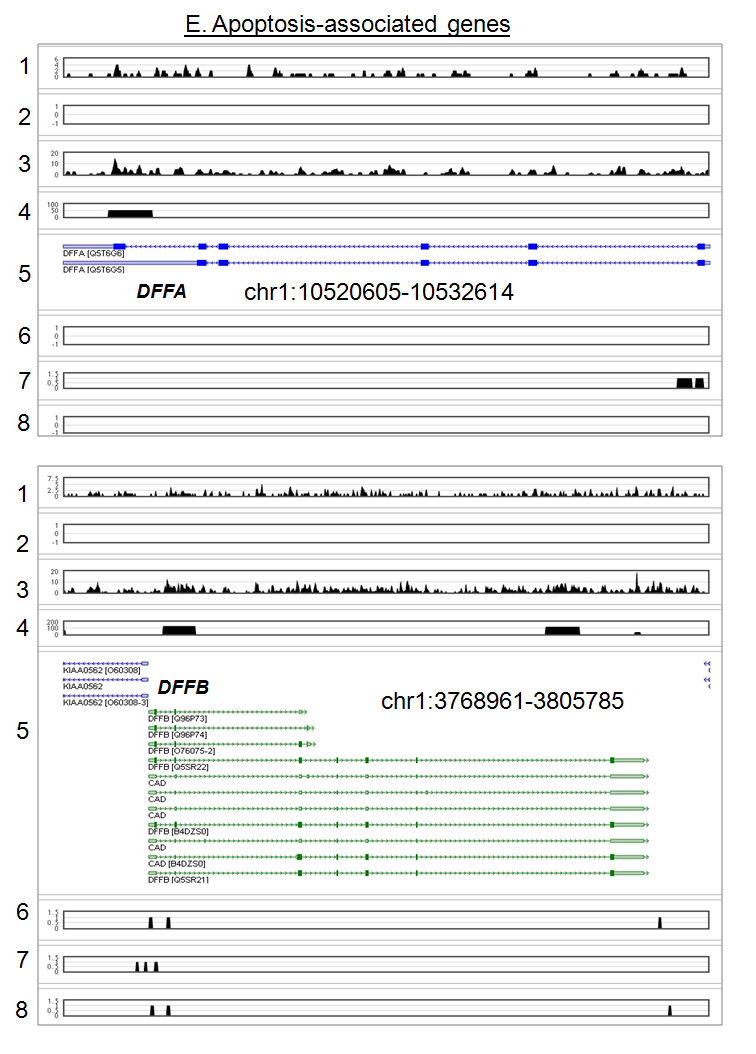
**

**Supplementary Figure 4.** **Screenshot examples**. E. Examples of regions with apoptosis-associated genes. Track labels follow labels in Figure 1B. (Continued from previous page).

**
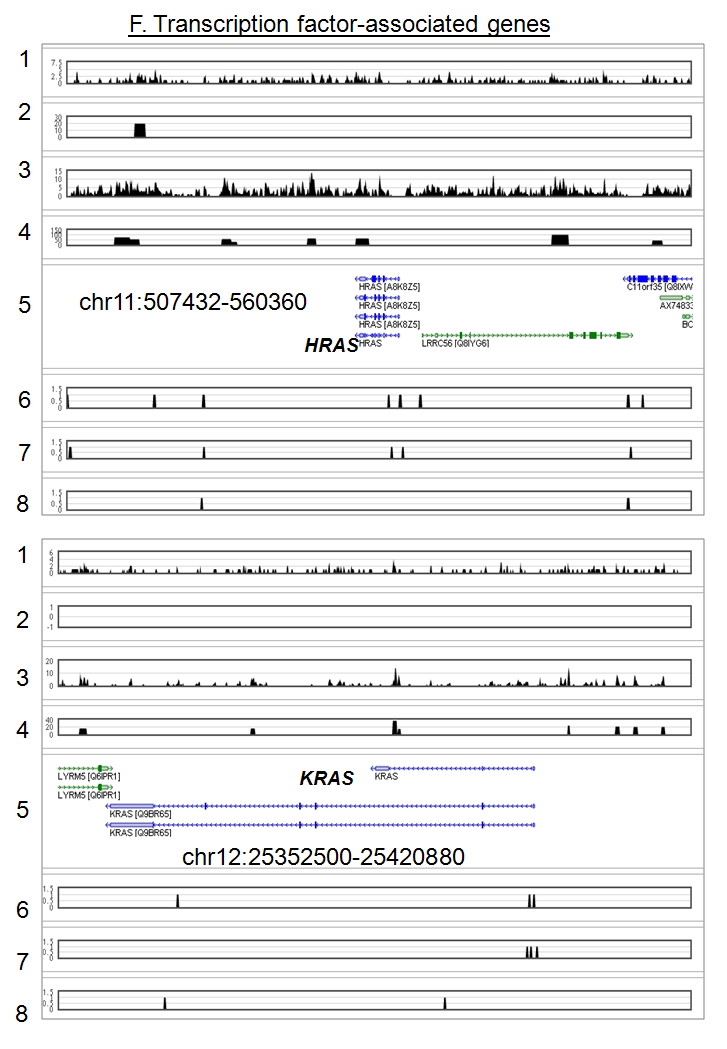
**

**Supplementary Figure 4.** **Screenshot examples**. F. Examples of regions associated with transcription factors. Track labels follow labels in Figure 1B.
