# Supplementary material for: Next-Generation Sequencing of Apoptotic DNA Breakpoints Reveals Association with Actively Transcribed Genes and Gene Translocations
Source: PLoS One. 2011 Nov 8;6(11):e26054. doi: 10.1371/journal.pone.0026054 (PMC3210745; doi:10.1371/journal.pone.0026054)
Supplement: Figure S5 — Apoptoseq validations. (DOC) [file pone.0026054.s005.doc]

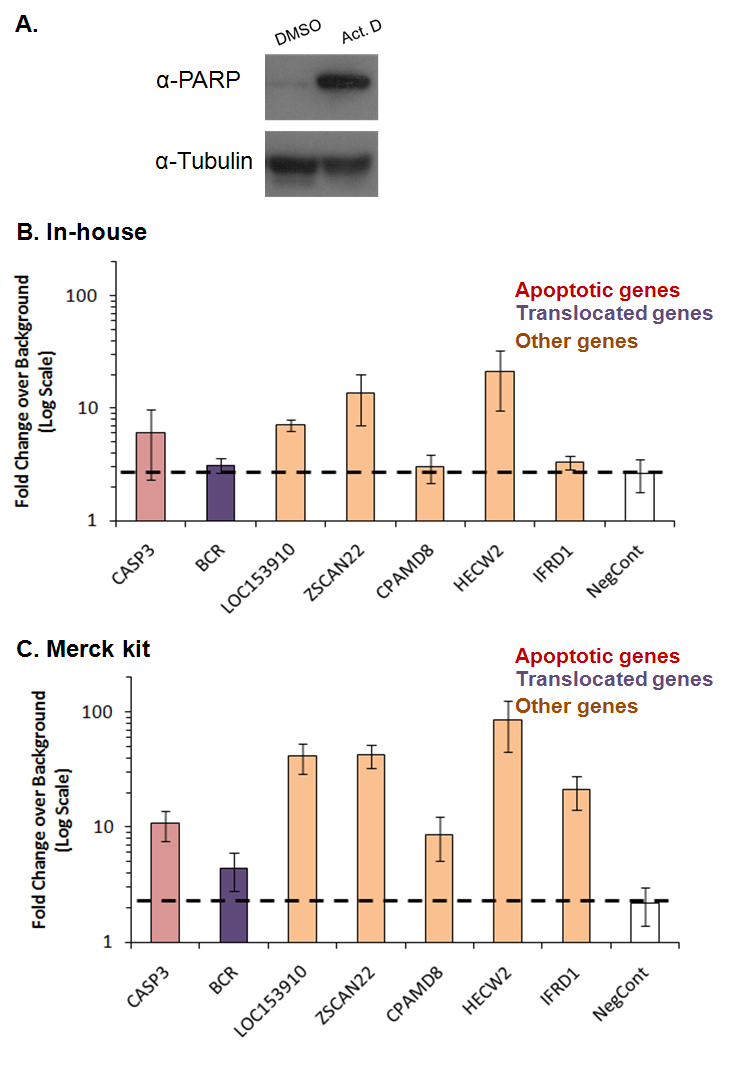


**Supplementary Figure 5. Apoptoseq validations.** A. Western immunoblotting of lysates from HL-60 cells treated with actinomycin D for 19h separated on SDS-PAGE is compared with ceels following treatment with vehicle (DMSO) and shows higher cleaved PARP levels. Tubulin immunoblotting was used to control for protein loading. 3 replicates were performed and a representative image is shown. B. Quantitative PCR validations were performed against apoptotic DNA ladders performed in-house (full results are shown in Figure 3A). C. Quantitative PCR validations were performed against apoptotic DNA ladders prepared from Merck Suicide-Track kit positive controls. The columns represent the average of three replicates. Error bars shown indicate s.e. The results shown in panel C are similar to the results in panel B.
