# Supplementary material for: Next-Generation Sequencing of Apoptotic DNA Breakpoints Reveals Association with Actively Transcribed Genes and Gene Translocations
Source: PLoS One. 2011 Nov 8;6(11):e26054. doi: 10.1371/journal.pone.0026054 (PMC3210745; doi:10.1371/journal.pone.0026054)
Supplement: Figure S6 — Analyses of apoptotic DNA breakpoints. (DOC) [file pone.0026054.s006.doc]

**
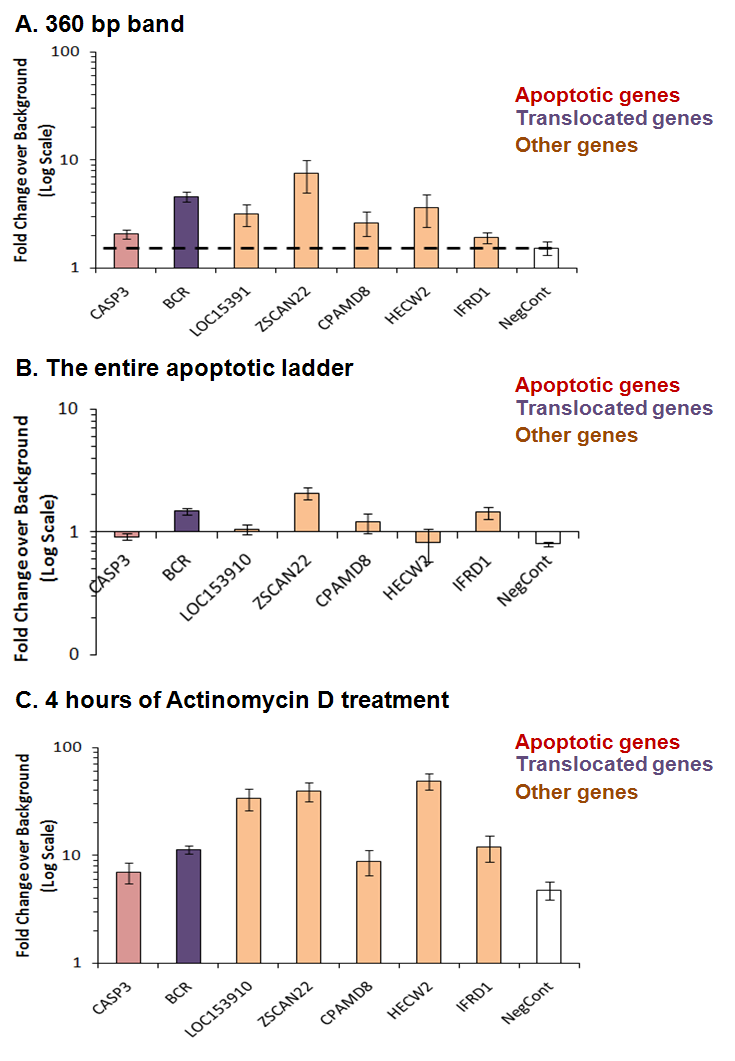
**

**Supplementary Figure 6. Analyses of apoptotic DNA breakpoints.** A. Analyses of 360 bp fragments from HL-60 cells treated with Actinomycin D for 19h indicates that these larger fragments shared some of the same apoptotic DNA breakpoints as found in 180 bp fragments. B. Analyses of the entire apoptotic ladder from HL-60 cells treated with Actinomycin D for 19h suggested that fragments larger than 360 bp displayed fewer apoptotic DNA breakpoints. C. Analyses of the 180 bp band from HL-60 cells treated with Actinomycin D for 4 hours indicates that apoptotic DNA cleavage was completed prior to 19 hours. The columns represent the average of three replicates. Error bars shown indicate s.e.
