# Supplementary material for: Next-Generation Sequencing of Apoptotic DNA Breakpoints Reveals Association with Actively Transcribed Genes and Gene Translocations
Source: PLoS One. 2011 Nov 8;6(11):e26054. doi: 10.1371/journal.pone.0026054 (PMC3210745; doi:10.1371/journal.pone.0026054)
Supplement: Figure S7 — Hypothesized model for apoptotic DNA fragmentation in cancer development. (DOC) [file pone.0026054.s007.doc]

**
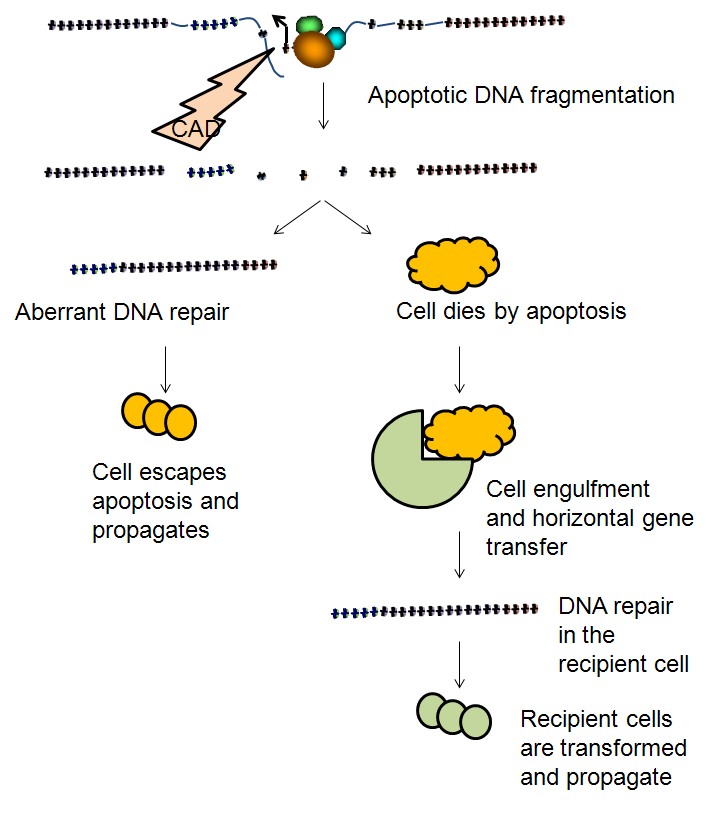
**

**Supplementary Figure 7. Hypothesized model for apoptotic DNA fragmentation in cancer development.** We have found that apoptosis, or possibly aberrant activation of CAD, leads to DNA fragmentation, particularly in open chromatin regions where active transcription may be ongoing. We hypothesize that the DNA may then undergo aberrant DNA repair, leading to translocations involving the DNA fragments, which may then confer the cell with a survival advantage, allowing the cell to escape apoptosis and propagate. Alternatively, the cell may die by apoptosis and the DNA may be passed on to the engulfing cell through horizontal gene transfer. The engulfing cell may then aberrantly integrate the DNA fragments into its genome through DNA repair, leading to translocations, which may then transform the engulfing cell into a cancer cell.
